# New Numerical Results from Simulations of Beams and Space Frame Systems with a Tuned Mass Damper

**DOI:** 10.3390/ma12081329

**Published:** 2019-04-23

**Authors:** Nguyen Chi Tho, Nguyen Tri Ta, Do Van Thom

**Affiliations:** 1 Institute of Techniques for Special Engineering, Le Quy Don Technical University, Ha Noi City 100000, Vietnam; chitho.mta@gmail.com (N.C.T.); nguyentrita@gmail.com (N.T.T.); 2 Faculty of Mechanical Engineering, Le Quy Don Technical University, Ha Noi City 100000, Vietnam

**Keywords:** space frame, tuned mass damper, finite element, random load, stationary process

## Abstract

In working processes, mechanical systems are often affected by both internal and external forces, which are the cause of the forced vibrations of the structures. They can be destroyed if the amplitude of vibration reaches a high enough value. One of the most popular ways to reduce these forced vibrations is to attach tuned mass damper (TMD) devices, which are commonly added at the maximum displacement point of the structures. This paper presents the computed results of the free vibration and the vibration response of the space frame system under an external random load, which is described as a stationary process with white noise. Static and dynamic equations are formed through the finite element method. In addition, this work also establishes artificial neural networks (ANNs) in order to predict the vibration response of the first frequencies of the structure. Numerical studies show that the data set of the TMD device strongly affects the first frequencies of the mechanical system, and the proposed artificial intelligence (AI) model can predict exactly the vibration response of the first frequencies of the structure. For the forced vibration problem, we can find optimal parameters of the TMD device and thus obtain minimum displacements of the structure. The results of this work can be used as a reference when applying this type of structure to TMD devices.

## 1. Introduction

There are many different ways to reduce vibrations of structures, such as reinforcing stiffeners into a structure to enhance its global stiffer, using special materials with high strengths, and adding TMD devices. Energy dissipation equipment such as TMD devices is used in engineering applications all over the world. There have been many studies on structures with energy dissipation equipment. The original invention of TMD was first proposed by Frahm [1], who strongly influenced Den Hartog [2], who presented classical optimum frequency and damping for a structure under a harmonic load. In the open TMD literature, Elias and Matsagar [3] briefly introduced TMD devices. Optimality criteria and specific TMD applications directly affected TMD efficiency in the reduction vibrations. For example, the authors in [4,5] presented an optimal data set of TMDs of high-rise buildings subjected to wind loads; the authors in [6] studied optimal TMDs for skyscrapers and considered uncertain parameters based on the Monte Carlo approach and Latin Hypercube Sampling. Kaynia and Veneziano [7] carried out a time history response of a one-degree-of-freedom system in two cases (with and without a TMD), where the structure was influenced by a series of historical earthquakes. Tuan and Shang [8] examined the mitigating influence of a TMD on the mechanical dynamic behaviour of a 101-floor building (in Taipei city) through numerical simulation and experiments, in which the structure was subjected to seismic excitation and wind loads. Domizio et al. [9] presented nonlinear dynamic responses of a 4-story steel frame with a TMD under wind loads and far-fault earthquakes. Krenk and Jan [10] investigated the optimal parameters of a TMD attached to a mechanical system with one degree of freedom, and the computed results showed that the influence of the difference between the exact optimal frequency tuning under random load and the classic tuning for harmonic load was not negligible. Fujino and Masato [11] employed a perturbation technique to optimize the parameters of TMDs subjected to different types of external loading. The results showed that this approach was only accurate when mass ratios were less than 0.02. Gerges and Vickery [12] studied a one-degree-of-freedom system equipped with a TMD under random white noise to obtain the optimum parameters of TMD, after which a design graph for the over-optimum-damped TMDs was shown. Sun and Jahangiri [13] employed an analytical model to obtain an optimum design formula of parameters for a TMD, which was added to an offshore wind turbine. The main purpose of their work was to control the vibration of the structure. Matteo et al. [14] used a tuned liquid column damper (TLCD) to control the seismic response of base-isolated structures. This work aimed to meet the optimal design of this type of TLCD device by using a straightforward direct approach. Bigdeli and Kim [15] studied the influence of damping to control vibration of a structure consisting of a TMD, a tuned liquid damper, and a tuned liquid column damper. The experiment results showed that the mass and damping of these devices were the most important factors in reducing vibration of the structure.

Regarding open literature about mechanical systems dealing with TMD devices, Lievens et al. [16] presented a robust optimization method for the design of a TMD, and uncertainties in the modal parameters were taken into account. Jiménez-Alonso and Sáez [17] proposed a motion-based design optimization approach in order to research the behavior of footbridges under uncertainty conditions. In [18], Tributsch and Adam examined the seismic performance of TMDs based on sets of recorded ground motion, and the effect of detuning on the stroke of the TMD and on the structural response was evaluated and quantified. Domenico and Ricciardi presented research on, respectively, the earthquake-resilient design of base-isolated buildings with a TMD in the basement [19] and the optimal design and seismic performance of a tuned mass damper inerter (TMDI) for structures with nonlinear base isolation systems [20]. They continued exploring the reduction of dynamic responses of base-isolated buildings by introducing a TMD in the basement, below the isolation floor where most of the earthquake-induced displacement demand is concentrated. For reduction vibration problems, Pietrosanti and his colleagues [21] concerned the optimal design and performance evaluation of a TMDI to reduce dynamic vibrations. Once again, Domenico and Ricciardi similarly studied the dynamic performance of base-isolated structures via TMD and TMDI devices [22] and an enhanced base isolation system equipped with an optimal TMDI [23]. Elias et al. [24] investigated the effectiveness of distributed multiple tuned mass dampers (d-MTMDs) for the multimode control of chimneys for along-wind response, where the response of a chimney is controlled by adding TMDs. Hashimoto and his coworkers [25] studied the reduction of responses in high-rise buildings subjected to wind loading by considering the effect of TMD devices.

This paper aims to present new numerical results about free vibration behaviors of beams and space frame systems. The artificial neural network (ANN) model is proposed to predict the fundamental frequencies of the structures. This work also presents the vibration response of beams under random load, which is described as a stationary process with white noise.

The body of this paper is divided into four main sections. Section 2 presents the finite element formulations for the space frame system with a TMD in the stationary process with white noise. A review of ANNs is presented in Section 3. Numerical results and discussions are given in Section 4, in which the ANN model is shown to predict the fundamental frequencies of the structure. In Section 5, important conclusions are summarized.

## 2. Geometry and Theoretical Formulations

### The Space Frame with a TMD

**Figure 1:**
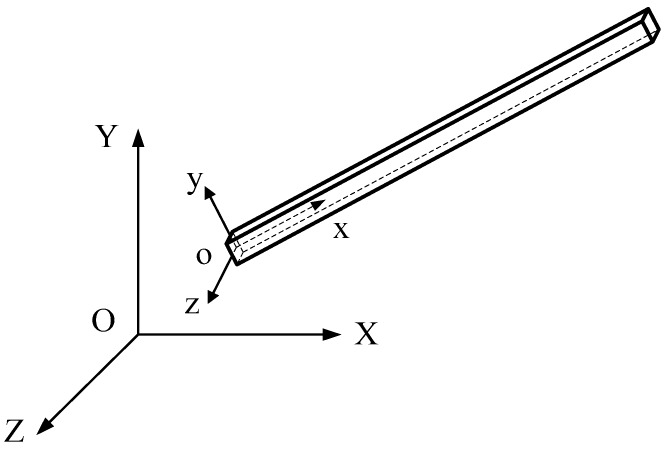
A model of the space frame element.

Consider a space frame element of the mechanical system (Figure 1). At any point on the frame elenment, there is a local coordinate *x* that has an unknown displacement vector **u**(*x*, *t*).

(1) $$u={\left[\begin{array}{cccccc} u & v & \theta_{z} & w & \theta_{y} & \varphi \end{array}\right]}^{T}$$

in which *u*, *v*, *w*, and *φ* are the displacments along the *x-*, *y-*, and *z*-directions and the twisting displacement around the *x*-axis, respectively. *θ_z_*, *θ_y_* are the angular components corresponding to the displacements *u* and *w*.

(2) $$\theta_{z}=\frac{dv}{dx};\theta_{y}=\frac{dw}{dx}.$$

Consider **q** is the nodal displacement of the frame element:

(3) $$q\left(t\right)={\left[\begin{array}{cccc} q_{u}\left(t\right) & q_{v}\left(t\right) & q_{w}\left(t\right) & q_{\varphi }\left(t\right) \end{array}\right]}^{T}$$

where

(4) $$\left\{ \begin{array}{l} q_{u}\left(t\right)={\left[\begin{array}{cc} u_{1} & u_{2} \end{array}\right]}^{T}\\ q_{v}\left(t\right)={\left[\begin{array}{cccc} v_{1} & \theta_{1z} & v_{2} & \theta_{2z} \end{array}\right]}^{T}\\ q_{w}\left(t\right)={\left[\begin{array}{cccc} w_{1} & \theta_{1y} & w_{2} & \theta_{2y} \end{array}\right]}^{T}\\ q_{\varphi }\left(t\right)={\left[\begin{array}{cc} \varphi_{1} & \varphi_{2} \end{array}\right]}^{T} \end{array}\right..$$

The displacement field of the frame element is approximated through the nodal displacement vector as follows

(5) u(x,t)=N(x)q(t)

where **N** are the shape functions

(6) $$N\left(x\right)=\left[\begin{array}{cccc} N_{u}\left(x\right) & & & \\ & N_{v}\left(x\right) & & \\ & & N_{w}\left(x\right) & \\ & & & N_{\varphi }\left(x\right) \end{array}\right].$$

Herein, the Hermite shape functions are employed and are expressed as follows:

(7) $$N_{u}\left(x\right)=\left[\begin{array}{cc} N_{1u}\left(x\right) & N_{2u}\left(x\right) \end{array}\right]$$

(8) $$N_{v}\left(x\right)=\left[\begin{array}{cccc} N_{1v}\left(x\right) & N_{2v}\left(x\right) & N_{3v}\left(x\right) & N_{4v}\left(x\right)\\ \frac{dN_{1v}\left(x\right)}{dx} & \frac{dN_{2v}\left(x\right)}{dx} & \frac{dN_{3v}\left(x\right)}{dx} & \frac{dN_{4v}\left(x\right)}{dx} \end{array}\right]$$

(9) $$N_{w}\left(x\right)=\left[\begin{array}{cccc} N_{1w}\left(x\right) & N_{2w}\left(x\right) & N_{3w}\left(x\right) & N_{4w}\left(x\right)\\ \frac{dN_{1w}\left(x\right)}{dx} & \frac{dN_{1w}\left(x\right)}{dx} & \frac{dN_{1w}\left(x\right)}{dx} & \frac{dN_{1w}\left(x\right)}{dx} \end{array}\right]$$

(10) $$N_{\varphi }\left(x\right)=\left[\begin{array}{cc} N_{1\varphi }\left(x\right) & N_{2\varphi }\left(x\right) \end{array}\right]$$

in which *a* is the length of the frame element. These shape functions can be found in the Appendix A.

The elastic potential energy of the internal forces in the frame element is calculated as follows:

(11) $$\begin{array}{ll} A= & \frac{1}{2}\{EF\int_{0}^{a}{\left[u^{\prime }\left(x,t\right)\right]}^{2}dx+EI_{z}\int_{0}^{a}{\left[v^{\prime \prime }\left(x,t\right)\right]}^{2}dx\\ & \;+EI_{y}\int_{0}^{a}{\left[w^{\prime \prime }\left(x,t\right)\right]}^{2}dx+GJ_{p}\int_{0}^{a}{\left[\varphi^{\prime \prime }\left(x,t\right)\right]}^{2}dx\} \end{array}$$

where $u^{\prime },\;v^{\prime \prime },\;w^{\prime },\;\;\varphi^{\prime }$ are the derivative components of *u*, *v*, *w*, and *φ* displacements, correspondingly. *E* is the elastic modulus of the material. *G* is the shear modulus of the material. *F* is the area of the cross-section of the frame element. *I_y_* and *I_z_* are the moments of inertia of the area in the *y-* and *z*-directions in the local coordinate system. *J_p_* is the polar moment of inertia of the area.

Note that Equations (5) and (11) can be rewritten in the matrix form as follows:

(12) $$A=\frac{1}{2}\int_{0}^{a}D{\left[B\left(x\right)q\left(t\right)\right]}^{2}dx=\frac{1}{2}q^{T}\left(t\right)\left[\int_{0}^{a}B^{T}\left(x\right)\mathrm{DB}\left(x\right)dx\right]q\left(t\right)$$

where **B** is the strain-displacement relation matrix of the frame element.

(13) $$B\left(x\right)=\left[\begin{array}{cccc} {N^{\prime }}_{u}\left(x\right) & & & \\ & {N^{\prime \prime }}_{v}\left(x\right) & & \\ & & {N^{\prime \prime }}_{w}\left(x\right) & \\ & & & {N^{\prime }}_{\varphi }\left(x\right) \end{array}\right]$$

**D** is the eslastic matrix of the frame element.

(14) $$D=\left[\begin{array}{cccc} EF & & & \\ & EI_{z} & & \\ & & EI_{y} & \\ & & & GJ_{p} \end{array}\right]$$

The work done by external forces is expressed as follows:

(15) $$\begin{array}{ll} A_{e} & =-\left\{\begin{array}{l} \int_{0}^{a}p_{u}(x,t)u(x,t)dx+\int_{0}^{a}p_{v}(x,t)v(x,t)dx+\int_{0}^{a}p_{w}(x,t)w(x,t)dx+\\ \int_{0}^{a}p_{\varphi }(x,t)\varphi (x,t)dx \end{array}\right\}+\\ & -\frac{1}{2}\left\{\begin{array}{l} \int_{0}^{a}-m\ddot{u}(x,t)u(x,t)dx+\int_{0}^{a}-m\ddot{v}(x,t)v(x,t)dx+\int_{0}^{a}-m\ddot{w}(x,t)\omega (x,t)dx+\\ \int_{0}^{a}-m_{p}\ddot{\varphi }(x,t)\varphi (x,t)dx \end{array}\right\}+\\ & -\frac{1}{2}\left\{\begin{array}{l} \int_{0}^{a}-c\dot{u}(x,t)u(x,t)dx+\int_{0}^{a}-c\dot{v}(x,t)v(x,t)dx+\int_{0}^{a}-c\dot{w}(x,t)w(x,t)dx+\\ \int_{0}^{a}-c_{p}\dot{\varphi }(x,t)\varphi (x,t)dx \end{array}\right\} \end{array}$$

Equation (15) can be rewritten in the matrix form as follows:

(16) $$\begin{array}{lll} A_{e} & = & -q^{T}\left(t\right)\int_{0}^{a}N^{T}\left(x\right)p\left(x,t\right)dx+\\ & & \;+\frac{1}{2}q^{T}\left(t\right)\int_{0}^{a}N^{T}\left(x\right)N\left(x\right)dxm_{e}\ddot{q}\left(t\right)\\ & & \;+\frac{1}{2}q^{T}\left(t\right)\int_{0}^{a}N^{T}\left(x\right)N\left(x\right)dxc\ddot{q}\left(t\right) \end{array}$$

where *m* is the mass per unit length. *m_p_* is the moment of inertia of *m* per unit length, which is defined as follows:

(17) $$m_{p}=mJ_{p}$$

**Figure 2:**
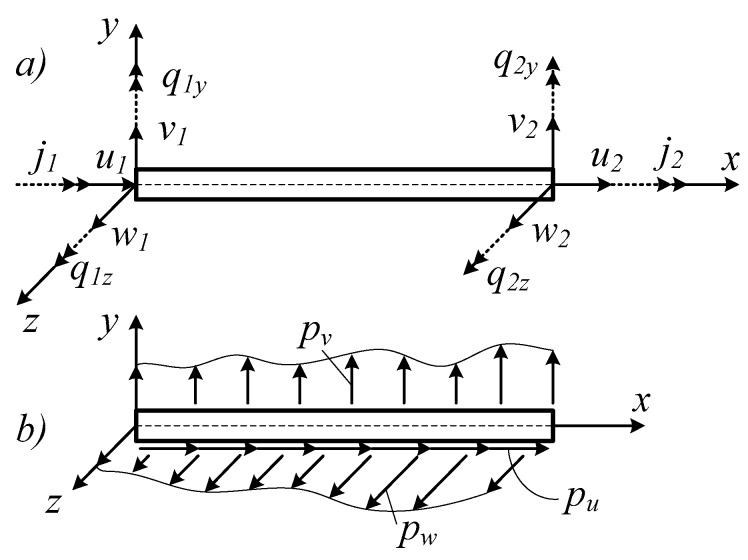
The displacement components at element nodes (**a**); the distributed load components on the frame element (**b**).

*c* is the viscous drag coefficient per unit length. *p_u_*, *p_v_*, *p_w_*, and *p_φ_* are the distributed loads per unit length (Figure 2b). **p**(*x*,*t*) is the distributed load on the frame element:

(18) $$p\left(x,t\right)={\left[\begin{array}{cccc} p_{u}\left(x,t\right) & p_{v}\left(x,t\right) & p_{w}\left(x,t\right) & p_{\varphi }\left(x,t\right) \end{array}\right]}^{T}.$$

**m***_e_* is the distributed mass matrix of the frame element:

(19) $$m_{e}=\left[\begin{array}{cccc} m_{u} & & & \\ & m_{v} & & \\ & & m_{w} & \\ & & & m_{\varphi } \end{array}\right]$$

in which distributed mass matrix components are given in the Appendix A.

According to the minimum of the potential energy, the equilibrium condition of the system has a form as follows:

(20) $$\frac{\partial V}{\partial q_{i}}=\frac{\partial \left(A+A_{e}\right)}{\partial q_{i}}=0\;\mathrm{with}\;i\;=\;u,\;v,\;w,\;\varphi .$$

Substituting Equations (12) and (16) into Equation (20) with generalized coordinates *i* are *u*, *v*, *w*, and *φ*, respectively, we obtain the finite element oscillation equation as follows:

(21) $$m\ddot{q}\left(t\right)+c\dot{q}\left(t\right)+kq\left(t\right)=p\left(t\right)$$

where **k**, **m**, and **c** are the element stiffness matrix, the element mass matrix, and the element viscous drag matrix, respectively.

(22) $$\begin{array}{l} k=\int_{0}^{a}B^{T}\left(x\right)DB\left(x\right)dx\\ m=m_{e}^{T}\int_{0}^{a}N^{T}\left(x\right)N\left(x\right)dx\\ c=c\int_{0}^{a}N^{T}\left(x\right)N\left(x\right)dx \end{array}$$

**p** is the element nodal force:

(23) $$p\left(t\right)=\int_{0}^{a}N{\left(x\right)}^{T}p\left(x,t\right)dx.$$

By integrating Equations (22) and (23), we obtain the finite element matrices of the space frame element. These element matrices are given in the Appendix A.

**Figure 3:**
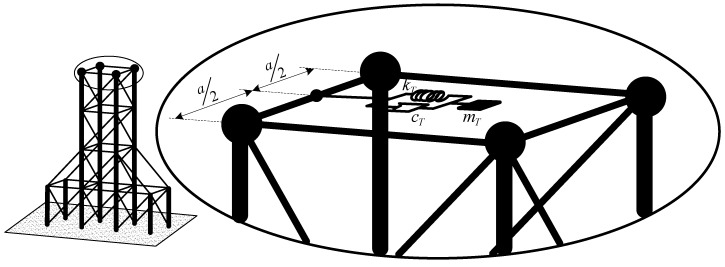
The real space frame system with a tuned mass damper (TMD).

We suppose a TMD is installed on the top horizontal bar of the frame system (Figure 3), assuming the TMD only transverses in the horizontal direction corresponding to displacement *w* of this system. Let *w_T_* be the displacement of the mass *m_T_* in the TMD vibration reduction system. We can then determine the vibration equation of the mass *m_T_* as the following formula:

(24) $$m_{T}{\ddot{w}}_{T}+c_{T}\left({\dot{w}}_{T}-{\dot{w}}_{0}\right)+k_{T}\left(w_{T}-w_{0}\right)=F\left(t\right)$$

where *m_T_*, *c_T_*, and *k_T_* are the mass, damper, and stiffness of the TMD, respectively. When adding the TMD, the concentrated force **f***_T_* due to TMD acting on the system is defined as follows:

(25) $$f_{T}=m_{T}g+k_{T}(w_{T}-w_{0})+c_{T}({\dot{w}}_{T}-{\dot{w}}_{0})$$

where

(26) $$w_{0}=N_{w}q;\;{\dot{w}}_{0}=\frac{\partial w_{0}}{\partial t}=N_{w}\dot{q}$$

By combining two vibration equations (Equations (21) and (24)), we obtain the dynamic equation of the structure with a TMD as follows:

(27) $$\left[\begin{array}{cc} m & 0\\ 0 & m_{T} \end{array}\right]\left\{\begin{array}{c} \ddot{q}\\ {\ddot{w}}_{T} \end{array}\right\}+\left[\begin{array}{cc} c+c_{T}N_{w}^{T}N_{w} & -c_{T}N_{w}^{T}\\ -c_{T}N_{w} & c_{T} \end{array}\right]\left\{\begin{array}{c} \dot{q}\\ {\dot{w}}_{T} \end{array}\right\}+\left[\begin{array}{cc} k+k_{T}N_{w}^{T}N_{w} & -k_{T}N_{w}^{T}\\ -k_{T}N_{w} & k_{T} \end{array}\right]\left\{\begin{array}{c} q\\ w_{T} \end{array}\right\}\;=\;\left\{\begin{array}{c} p\left(t\right)\\ F\left(t\right) \end{array}\right\}$$

When establishing finite element matrices of elements, each element is set in a local coordinate system (called the element coordinate system). The components of the element nodal force vector and the element nodal displacement vector are selected correspondingly to the directions of this coordinate system. When setting up the motion equation of the structure, the above vectors need to be converted to the global coordinate system of the structure.

The relation of the force vector and the displacement vector in the local coordinate and the global coordinate is described as follows:

(28) $$q=T\bar{q}$$

in which **q** is the displacement vector or the load vector in the local coordinate system. $\bar{q}$ is the displacement vector or the load vector in the global coordinate system. **T** is the transformation matrix between vectors **q** and $\bar{q}$. The relationship of the nodal displacement components between the local coordinate system and the global coordinate system of the structure is given in the Appendix A.

Therefore, the equation of forced vibration of the frame element with a TMD has the following form.

(29) $$\left[\begin{array}{cc} T^{T}\mathrm{mT} & 0\\ 0 & m_{T} \end{array}\right]\left\{\begin{array}{c} \ddot{\bar{q}}\\ {\ddot{w}}_{T} \end{array}\right\}+\left[\begin{array}{cc} T^{T}\left(c+c_{T}N_{w}^{T}N_{w}\right)T+ & -c_{T}N_{w}^{T}T\\ -c_{T}N_{w}T & c_{T} \end{array}\right]\left\{\begin{array}{c} \bar{q}\\ {\dot{w}}_{T} \end{array}\right\}+\left[\begin{array}{cc} T^{T}\left(k+k_{T}N_{w}^{T}N_{w}\right)T+ & -k_{T}N_{w}^{T}T\\ -k_{T}N_{w}T & k_{T} \end{array}\right]\left\{\begin{array}{c} \bar{q}\\ w_{T} \end{array}\right\}\;=\;\left\{\begin{array}{c} T^{T}p\left(t\right)\\ F\left(t\right) \end{array}\right\}.$$

By assembling the element matrices and vectors and eliminating boundary conditions, we obtain the forced oscillation equation of the structure as follows:

(30) $$M\ddot{Q}+C\dot{Q}+\mathrm{KQ}=F$$

where **M, C,** and **K** are the global mass matrix, the global viscous drag matrix, and the global stiffness matrix of the structure, respectively, and **Q** and **F** are the global displacement vector and the global force vector of the structure, respectively.

For the free vibration without the viscous drag, Equation (30) becomes

(31) $$M\ddot{Q}+\mathrm{KQ}=0.$$

We then obtain the equation to determine the natural frequencies and the mode shapes:

(32) $$\left(K-\omega^{2}M\right)Q=0.$$

Consider a mechanical system under random loads with the forced vibration equation as Equation (30); the random load is described as a stationary process with the average value as follows:

(33) $$m_{F}={\left\{\begin{array}{cccc} m_{F_{1}} & m_{F_{2}} & \ldots & m_{F_{n}} \end{array}\right\}}^{T}.$$

The correlation matrix is

(34) $$R_{F}\left(\tau \right)=\langle F\left(t\right)F\left(t+\tau \right)\rangle$$

where $R_{F_{i}F_{j}}=\langle F_{i}\left(t\right)F_{j}\left(t+\tau \right)\rangle$ is the correlation between two functions **F**_*i*_ and **F**_*j*_ at two moments *t* and (t+τ).

In order to obtain the average value of the response, taking the average of two sides of Equation (30), we obtain

(35) $$M\langle \ddot{Q}\rangle +C\langle \dot{Q}\rangle +K\langle Q\rangle =\langle F\rangle .$$

Because of the assumption that *Q* is the stationary process (〈Q〉 is constant), we have

(36) $$\langle \dot{Q}\rangle =\frac{d}{dt}\langle Q\rangle =0;\;\langle \ddot{Q}\rangle =\frac{d^{2}}{dt^{2}}\langle Q\rangle =0$$

Thus, from Equation (35), we obtain

(37) $$K\langle Q\rangle =\langle F\rangle \;\mathrm{or}\;\langle Q\rangle =K^{-1}\langle F\rangle .$$

The image equation of Equation (30) through the Fourier transform is

(38) $$M{\left(i\omega \right)}^{2}Q\left(\omega \right)+Mi\omega Q_{0}-{M\dot{Q}}_{0}+Ci\omega Q\left(\omega \right)-{\mathrm{CQ}}_{0}+\mathrm{KQ}\left(\omega \right)=F\left(\omega \right)$$

or

(39) $$\left(-{\left(i\omega \right)}^{2}M+i\omega C+K\right)Q\left(\omega \right)+\left(Mi\omega Q_{0}-{M\dot{Q}}_{0}-{\mathrm{CQ}}_{0}\right)=F\left(\omega \right).$$

When *t* = 0, $Q_{0}=0$ and ${\dot{Q}}_{0}=0$, we have

(40) $$\left(Mi\omega Q_{0}-{M\dot{Q}}_{0}-{\mathrm{CQ}}_{0}\right)=0.$$

Thus, from Equation (39), we have

(41) $$Q\left(\omega \right)=\left(-{\left(i\omega \right)}^{2}M+i\omega C+K\right)F\left(\omega \right)=H\left(\omega \right)F\left(\omega \right)$$

where

(42) $$H\left(\omega \right)={\left(-{\left(i\omega \right)}^{2}M+i\omega C+K\right)}^{-1}$$

is the transfer function matrix of the system.

Let $S_{FF}\left(\omega \right)$ be the spectral matrix of the input with the components $S_{F_{i}F_{j}}\left(\omega \right)$, H(ω) be the transfer function matrix of the system with the components $H_{ij}\left(\omega \right)$, and $S_{QQ}\left(\omega \right)$ be the spectral vector of function **Q** with its components $S_{Q_{i}Q_{j}}\left(\omega \right)$. We can then define the relation equation among the spectral matrices of the input $S_{QQ}\left(\omega \right)$, $S_{FF}\left(\omega \right)$, and the transfer function matrix H(ω).

The autocorrelation matrix of the response vector **Q** is

(43) $$R_{QQ}\left(\tau \right)=E\left({\mathrm{QQ}}^{T}\right).$$

Replacing **Q** with Duhamel’s integrals for multiple degrees of freedom system, we have

(44) $$\begin{array}{ll} R_{QQ}\left(\tau \right) & =E\left(\int_{-\infty }^{+\infty }h\left(\tau_{1}\right)F\left(t-\tau_{1}\right)d\tau_{1}\int_{-\infty }^{+\infty }F^{T}\left(t+\tau -\tau_{2}\right)h^{T}\left(\tau_{2}\right)d\tau_{2}\right)\\ & =\int_{-\infty }^{+\infty }\int_{-\infty }^{+\infty }h\left(\tau_{1}\right)h^{T}\left(\tau_{2}\right)E\left(F\left(t-\tau_{1}\right)F^{T}\left(t+\tau -\tau_{2}\right)\right)d\tau_{1}d\tau_{2} \end{array}$$

Because $E\left(F\left(t-\tau_{1}\right)F^{T}\left(t+\tau -\tau_{2}\right)\right)=R_{FF}\left(t+\tau_{1}-\tau_{2}\right)$,

(45) $$R_{QQ}\left(\tau \right)=\;\int_{-\infty }^{+\infty }\int_{-\infty }^{+\infty }h\left(\tau_{1}\right)h^{T}\left(\tau_{2}\right)R_{FF}\left(t+\tau_{1}-\tau_{2}\right)d\tau_{1}d\tau_{2}.$$

From the Wiener–Khinchin function, we have

(46) $$S_{QQ}\left(\omega \right)=\frac{1}{2\pi }\int_{-\infty }^{+\infty }R_{QQ}\left(\tau \right)e^{-i\omega t}d\tau .$$

Substituting Equation (45) into Equation (46), we obtain the matrix of spectral density functions of response as follows:

(47) $$\begin{array}{ll} S_{QQ}\left(\tau \right) & =\frac{1}{2\pi }\int_{-\infty }^{+\infty }\int_{-\infty }^{+\infty }\int_{-\infty }^{+\infty }h\left(\tau_{1}\right)h^{T}\left(\tau_{2}\right)R_{FF}\left(\tau +\tau_{1}-\tau_{2}\right)e^{-i\omega t}d\tau_{1}d\tau_{2}d\tau \\ & \;=\;\frac{1}{2\pi }\int_{-\infty }^{+\infty }\int_{-\infty }^{+\infty }h\left(\tau_{1}\right)h^{T}\left(\tau_{2}\right)\left(\int_{-\infty }^{+\infty }R_{FF}\left(\tau +\tau_{1}-\tau_{2}\right)e^{-i\omega t}d\tau \right)d\tau_{1}d\tau_{2} \end{array}$$

Let $\tau +\tau_{1}-\tau_{2}=\tilde{\tau }$, $\tau =\tilde{\tau }-\tau_{1}+\tau_{2}$ and we have

(48) $$S_{QQ}\left(\tau \right)=\frac{1}{2\pi }\int_{-\infty }^{+\infty }h\left(\tau_{1}\right)e^{i\omega \tau_{1}}d\tau_{1}\int_{-\infty }^{+\infty }h^{T}\left(\tau_{2}\right)e^{-i\omega \tau_{2}}d\tau_{2}\int_{-\infty }^{+\infty }R_{FF}\left(\tilde{\tau }\right)e^{-i\omega \tilde{\tau }}d\tilde{\tau }.$$

The first integral is

(49) $$\int_{-\infty }^{+\infty }h\left(\tau_{1}\right)e^{i\omega \tau_{1}}d\tau_{1}=H^{*}\left(\omega \right).$$

The second integral is

(50) $$\int_{-\infty }^{+\infty }h\left(\tau_{2}\right)e^{-i\omega \tau_{2}}d\tau_{2}=H^{T}\left(\omega \right).$$

The third integral is

(51) $$\int_{-\infty }^{+\infty }R_{FF}\left(\tilde{\tau }\right)e^{-i\omega \tilde{\tau }}d\tilde{\tau }=2\pi S_{FF}\left(\omega \right).$$

Finally, we have

(52) $$S_{QQ}\left(\omega \right)=H^{*}\left(\omega \right)S_{FF}\left(\omega \right)H^{T}\left(\omega \right).$$

Since this is a stationary process, the area of the spectral density function in the frequency domain is the variance of the response function. With the spectral density function of the constant agitation function, it is assumed that the white noise agitation, for each data set of the TMD, will make the density response function different.

## 3. Artificial Neural Networks

In recent years, along with the development of science and technology, applying artificial intelligence (AI) in solving complicated issues in science and in mechanical problems is important. The ANN model is based on a simulation of the working processes of the human brain. Neural network nodal functions can process numerous duties at the same time in a short amount of time to solve problems. A neural network is like a black box that can predict output data from a particular input. After a training process, the neural network can become aware of similarities from new input patterns [26]. There have been many science publications about the use of AI in analyzing mechanical behaviors, including design of experiments [27], genetic algorithms [28], ant colony optimization [29], fuzzy logic [30], and finite elements analysis [31]. Mohsen and Mazahery [32] employed a standard feed-forward network with a hidden layer. The number of neurons in both the input and output layers was defined by the variation data, and the optimal design was found through the numbers of neuron in the hidden layer and the mean square error. Esmailzadeh and Khafri [33] used finite element and artificial neural network to simulate a process of equal-channel angular pressing of an aluminum alloy. In [34], Abambres et al. introduced a neural network-based formula for the buckling load prediction of I-section cellular steel beams in order to precisely compute the critical buckling load of simply supported beams subjected to uniform loads. In mechanical systems, problems such as those related to resonance or the need to predict cracks require natural frequencies of the structure, and natural frequencies of the system are processed. Based on ANN models, we can predict the natural frequencies of the mechanical structures through the input data. Operating the ANN model costs less time and memories, and it can shorten the simulating time needed to export output parameters (in this work, the natural frequencies). This is one of the basic advantages of ANN models; they can be fully applied to other complex problems in mechanics.

## 4. Numerical Results and Discussions

### 4.1. Numerical Results for Free Vibration Analysis of the Beam and Frame System with a TMD

#### 4.1.1. Accuracy Studies

**Figure 4:**
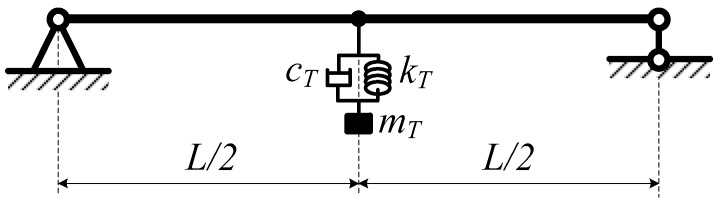
Beam with one TMD.

Firstly, a plane beam (2-dimensional beam) with a TMD attached at the mid-point is considered for the accuracy problem (Figure 4). The author compares the natural frequencies between this work and other exact publications. Note that the 2-dimensional beam only bends in one plane. At this time, each node retains three displacement components *u*, *w*, and $\theta_{y}$, which means each node has three degrees of freedom. Therefore, in order to obtain the element mass matrix, the element stiffness matrix in this plane, in the stiffness matrix and mass matrix of the space frame element above, we only need to remove rows and columns corresponding to the remaining degrees of freedom.

Consider a fully simply supported plane beam with the geometrical and material properties as follows: length = 1 m, width = 3 cm, height = 2 cm, density *ρ* = 7800 kg/m^3^, and Young’s modulus *E* = 2 × 10^11^ N/m^2^. The data set of the TMD is as follows [35]: *m_T_* = 0.468 kg, *k_T_* = 27,058.08 N/m, and *c_T_* = 100 Ns/m. The first natural frequency calculated by Hartog’s method [35] is 33.55 Hz, and the one of this work is 33.62 Hz. We understand that, although we employ different theories and methods, the results are still in good agreement, so the proposed theory and method in this work are verified.

#### 4.1.2. Numerical Results for Free Vibration Analysis of the Plane Beam with a TMD

Consider a fully simply supported beam with a TMD attached at the mid-point (Figure 4), where length *L* = 20 m, *EJ* = 2.66 × 10^7^ Nm^2^, density *ρ* = 7800 kg/m^3^ (*b* = *h* = 0.2 m, *E* = 2 × 10^11^ N/m^2^), and the total mass of the beam *m*_0_ = 6240 kg. For the TMD, *c_T_* = 100 Ns/m, and *k_T_* = 2000 N/m. The mass of the TMD *m_T_* varies such that its value is in a range of 1–10%*m*_0_. The first three natural frequencies are presented in Table 1. For the case where *m_T_* = 10%*m*_0_, *k_T_* = 2000 N/m, and the drag coefficient *c_T_* = 10–1000 Ns/m, the first three natural frequencies are presented in Table 2. For the case where *m_T_ =* 10%*m_0_*, *c_T_ =* 100 Ns/m, and the stiffness of the TMD *k_T_ =* 100*–*10,000 N/m, the first three natural frequencies are presented in Table 3.

**Figure 5:**
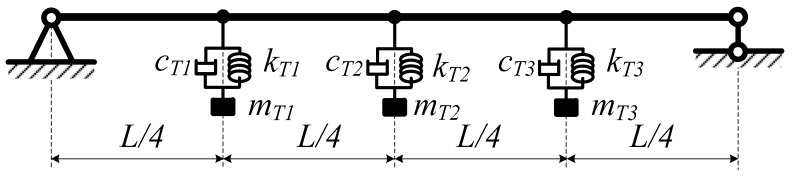
Beam with three TMDs.

In the case of attaching TMDs at three positions (Figure 5), one at the mid-point and the other two at positions such that the distance from there to the first one is *L*/4, the mass of the first TMD is equal to the third, *m_T_*_1_ = *m_T_*_3_. By varying the masses of TMDs so that the total mass of the three TMDs is not larger than 10%*m*_0_, the first three natural frequencies are presented in Table 4. In the case where *m*_T1_ = *m*_T2_ = *m*_T3_ = 3%*m*_0_, where *c*_T1_ = 10–1000 Ns/m and *k*_T1_ = 2000 N/m (*c*_T1_ = *c*_T2_ = *c*_T3_, and *k*_T1_ = *k*_T2_ = *k*_T3_), the first three natural frequencies are presented in Table 5. Finally, in the case where *c*_T1_ = 100 Ns/m, and *k*_T1_ = 100–10,000 N/m, the first three natural frequencies are presented in Table 6.

##### The Beam with One TMD

When increasing the mass of the TMD, the first natural frequency decreases, and the other natural frequencies are almost not changed despite this increase. When the mass of the TMD reaches 1% of the mass of the beam, the first natural frequency of the structure with one TMD is equal to 76.40% of the beam without a TMD (meaning this frequency is reduced by 23.60%). The frequency reduction rate, which can be up to 75.62%, increases when the mass of the TMD increases, indicating that a TMD has a strong effect on the first natural frequency of the structure.

When *m_T_* is equal to 10% of the total mass of the structure, the stiffness of the spring *k* is constant. When the damper of the TMD increases from 10 to 1000 Ns/m, the first natural frequencies are not changed. For this case, we can state that the viscous drag coefficient has a light influence on the free vibration of the structure.

However, when *m*_2_ is equal to 10% of the total mass of the structure, the drag coefficient of the TMD remains at 100 Ns/m. When the stiffness of the spring *k* increases from 10 to 10,000 N/m, the first natural frequency of the beam also increases. When the damper of the TMD is equal to 100 Ns/m, the stiffness of the spring *k_T_* is equal to 100 N/m; the first natural frequency of the beam can be reduced by 94.57%.

##### The Beam with Three TMDs

When the masses of TMD1 and TMD2 are equal to each other, increasing the mass of TMD2 reduces the first natural frequency of the structure. Similarly, the first natural frequency of the structure also decreases when the mass of TMD2 is constant, while the masses of TMD1 and TMD2 increase. When the total mass of the TMDs is equal to 10% of the mass of the structure, the first natural frequency only decreases by 61.50% (while the structure with one TMD can be up to 75.62%).

When the total mass of the TMDs is equal to 9% of the mass of the beam (each TMD obtains 3%), the stiffness of the spring *k_T_*_1_ (= *k_T_*_2_ = *k_T_*_3_) is 2000 N/m. When the damper of the TMD increases from 10 to 1000 Ns/m (*c_T_*_1_ = *c_T_*_2_ = *c_T_*_3_), the first natural frequencies are almost not changed (the phenomenon is similar to that of the structure with one TMD at the mid-point).

When the total mass of TMDs is equal to 9% of the mass of the beam (each TMD obtains 3%), the damper is a constant 100 Ns/m (*c_T_*_1_ = *c_T_*_2_ = *c_T_*_3_). The stiffness of the spring *k_T_*_1_ (= *k_T2_ = k_T_*_3_) increases in a range of 100–10,000 N/m, and the first natural frequencies of the structure increase. This phenomenon is also similar to that of the structure with one TMD at the mid-point. This time, the first natural frequency is decreased by 90%.

### 4.2. Numerical Results for Free Vibration Analysis of the Space Frame System with a TMD

**Figure 6:**
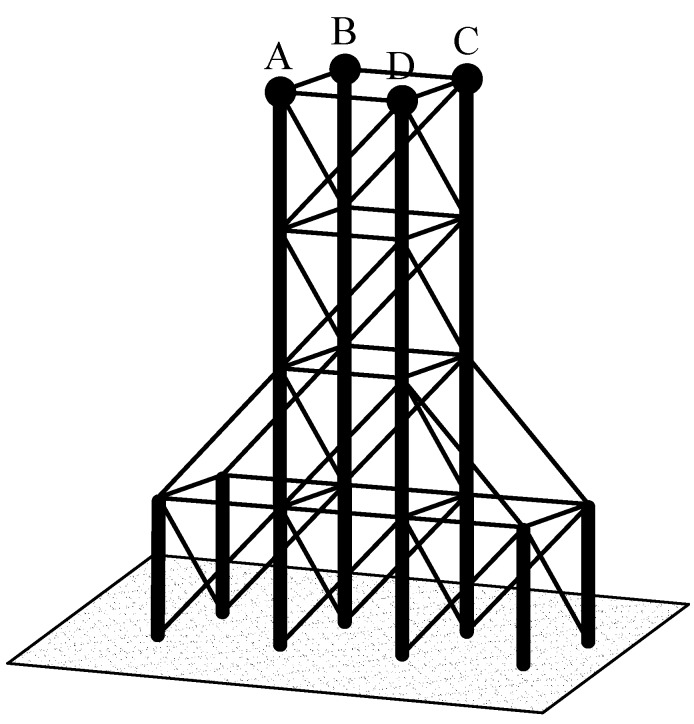
The real space frame system without a TMD.

Consider a mechanical space frame system as shown in Figure 6, in which the frame elements are made from the different steel straight pipes. Herein, we have two types of frame elements: (1) vertical frames where outer diameter *D*_0_ = 0.5 m, the area of the cross-section *F_0_* = 1.57 × 10^−3^ m^2^, bending moments of inertia *J_x0_* = *J_z0_* = 4.88 × 10^−5^ m^4^, and the polar moment of inertia *J_p0_* = 9.76 × 10^−5^ m^4^; (2) frames where *D* = 0.3 m, *F* = 9.40 × 10^−2^ m^2^, *J_x_* = *J_z_* = 1.05 × 10^−5^ m^4^, and *J_p_* = 2.1 × 10^−5^ m^4^. The mechanical properties of the steel are as follows: Young’s modulus *E* = 2 × 10^11^ N/m^2^, shear modulus *G* = 0.8 × 10^11^ N/m^2^, and density *ρ* = 7850 kg/m^3^. The concentrated loads of 4 × 27,000 kg are subject to four top points (A, B, C, and D). The total height of the frame system is 20 m. The results of the first three natural frequencies of this work, obtained from commercial software SAP-2000, are presented in Table 7. We can see that the results are in good agreement, so the proposed theory and method are reliable.

When a TMD is added to the mid-point of the top horizontal frame (Figure 3), the total mass of the system is *m_0_* = 1.114 × 10^5^ kg. When the mass *m_T_*, the stiffness of the spring, and the damper *c_T_* of the TMD are changed, we obtain the results of the first three natural frequencies of the structure with and without a TMD as shown in Table 8, Table 9 and Table 10. 

When the mass of the TMD increases, the natural frequencies of the system decrease. The mass of the TMD has a strong effect on the first natural frequency of the structure. Two other natural frequencies are slightly affected by the mass of the TMD.

Similarly, the stiffness of the TMD has a strong influence on the first natural frequency. Two other natural frequencies are almost not changed when the stiffness of the TMD increases.

Finally, increases in the damper of the TMD have a light effect on the first three natural frequencies. The values of the first three natural frequencies are almost not varied by the damper in the exploring domain.

### 4.3. Setup of the ANN Model

**Figure 7:**
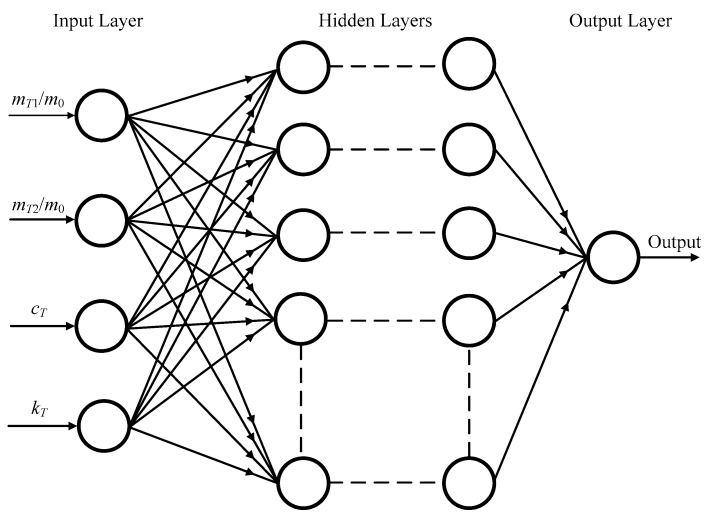
The ANN model with four inputs and one output.

**Figure 8:**
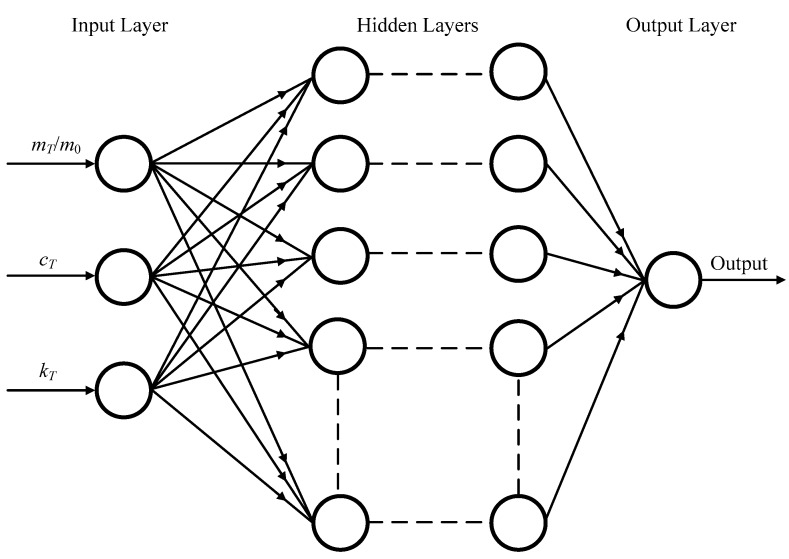
The ANN model with three inputs and one output.

We first chose the training set, which are the parameters of the TMDs and the fundamental frequencies; for the beam with three TMDs, the ANN model has four data inputs: *m_T_*_2_/*m*_0_, *m_T_*_1_/*m*_0_, *c_T_* (*c_T_* = *c_T_*_1_ = *c_T_*_2_ = *c_T_*_3_), and the stiffness *k_T_* (*k_T_* = *k_T_*_1_ = *k_T_*_2_ = *k_T_*_3_) of the TMD, and the one data output is the fundamental frequency (see Figure 7). In this model, 27 randomly selected data are given to train the network (from a total of 32), and the other five data are test data. Similarly, for the space frame system with one TMD, the ANN model has three data inputs *m_T_*/*m*_0_, *c_T_*, and *k_T_* of the TMD, and the one data output is the fundamental frequency. There are 22 randomly selected data (from a total of 26) and 4 four data for training and testing, respectively (see Figure 8). In this work, the proposed model is designed and computed in a MATLAB environment. The quantifiable method cannot be used to evaluate the best network architecture, so we seek to optimize the verification of the ANN model. A text spreadsheet input file includes both the training data input and the output for the training stage. One to three hidden number layers are chosen in order to discover the number of layers required to model the process. In each one of the hidden layers, the number of nodes (neurons) is changed in a range of 4–80 neurons. To control the magnitude of the weight and bias updates, the learning rate parameter is set during the simulation process. Therefore, the training time of the ANN depends strongly on the selection of this value. In a MATLAB environment, the weights are automatically corrected after each case of the training data. In addition, one parameter (the momentum value) is employed to reduce the likeliness of the simulation, which can be stuck in local optima. Moreover, the learning rate is commonly established in a range from 0.0001 to 6.0, which depends on the simulation during the training process, and the momentum in the ANN model is retained at an average value of 0.8. In order to minimize the training error and avoid over training, the training process needs to be supervised. After testing a variety of hidden layer variations, we find that a hidden layer that includes six nodes achieves the most accurate prediction of frequencies. The computed and predicted values of the frequencies are listed in Table 11. The bold values are accurately predicted values. The average errors are presented in the penultimate row of Table 11, in which the percentage error (*e_i_*) is determined as the following formula:

(53) $$e_{i}=100\times \frac{\left(\mathrm{predict}\;\mathrm{value}-\mathrm{computed}\;\mathrm{value}\right)}{\mathrm{computed}\;\mathrm{value}}\%.$$

The absolute maximum percentage errors are presented in the bottom row of Table 11, $E_{\infty }$, which show the worst prediction errors of the model. In this case, we need the smallest values ($E_{\infty }$) in order to obtain the best predictions. Herein, $E_{\infty }$ can be defined as follows:

(54) $$E_{\infty }=\max_{i=1}^{n}\left(e_{i}\right).$$

From Table 11 and Table 12, we can see that the maximum errors ($E_{\infty }$) are very small; $E_{\infty }$ = 0.7484% for the beam, and $E_{\infty }$ = 0.3052% for the space frame system. These computed results demonstrate that this ANN model can well predict the fundamental frequencies of the structure. This is the highlight of this work: the basis on which mechanical behavior predictions can be applied to many complicated problems in mechanics. Complicated mechanical systems require a great deal of work, and the use of ANN models can lead to precise results. There is no need to conduct calculations for the structure from the beginning, thus increasing simulation and calculation efficiency. In other words, we can apply the proposed ANN model to other models that only need input parameters, specific targets, the number of training data, and testing data. By selecting appropriately the number of nodes (neurons) in the hidden layers and the momentum, we can completely predict the desired results with acceptable errors in comparison with the original goals. For example, consider a plate with cracks, where each data set of plate parameters such as the length of the structure, the deviational angle of the crack, the location of the crack in the plate, and the changing laws of external loads are input into the data set. The output parameters are the growing lines of the cracks. Thus, by employing the ANN model, we can fully train each situation of the input data set to predict the development of the crack. Therefore, this is a method of great significance in technology; at this time; we do not need to run repeatedly the problem of dynamic crack propagation when facing a specific situation in practice (note that the simulation of a dynamic crack propagation problem, which requires a high-speed computer, is a relatively complex problem and is a waste of time). Therefore, we only need to import the input data set instead. The ANN model can output instant results regarding the development of the growing line of the crack, so engineers can handle arising matters as quickly as possible.

### 4.4. Numerical Results for Vibration Analysis of Beam with a TMD under Random Loading

**Figure 9:**
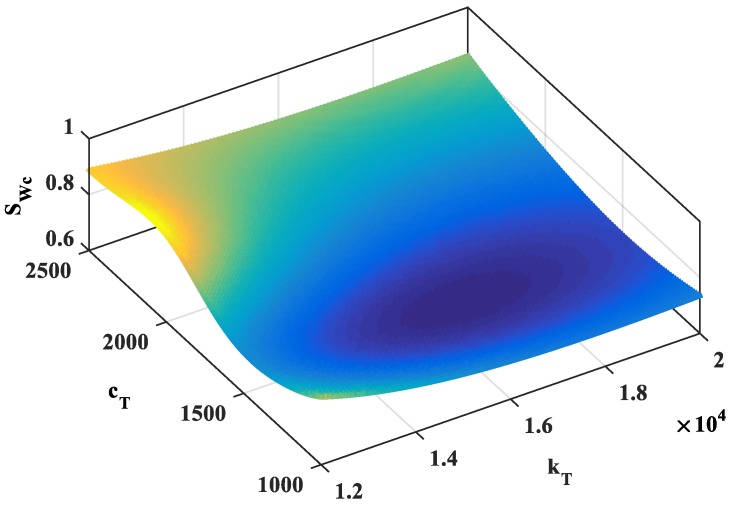
The variation of *S_Wc_* as a function of *c_T_* and *k_T_*.

**Figure 10:**
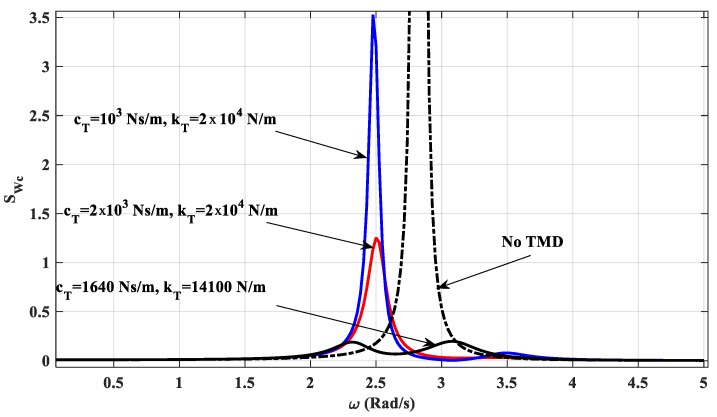
The spectral density function in the frequency domain.

Consider a fully simply supported beam where length *L* = 57 m, density μ= 54 kg/m, *EJ* = 6.384 × 10^8^ Nm^2^, and total mass of the beam *m*_0_ = 42,978 kg. We explore the first natural frequency of the beam without a TMD, which is 2.80 Hz. For the beam with one TMD, at the mid-point, the mass of the TMD *m_T_* is 5% of *m*_0_ (Figure 4); the beam is under a uniform random load (as a stationary process) where the white noise domain $S_{FF}\left(\omega \right)$ = 1. For each data set of the damper *c_T_* and the stiffness of the spring *k_T_* of the TMD, the mechanical responses of the structure are different. We will plot the response function $S_{Q_{i}Q_{j}}\left(\omega \right)$ of the degrees of freedom in the exploring frequency domain. The area limited by the response function in this frequency domain is the variance of response ($D_{Q_{i}Q_{j}}$) and is also the square of the standard deviation ${\left(\sigma_{Q_{i}Q_{j}}^{}\right)}^{2}.$ The standard deviation ${\left(\sigma_{Q_{i}Q_{j}}^{}\right)}^{2}$ changes as a function of *c_T_* and *k_T_*, as presented in Figure 9. From this figure, t can be seen that the minimum value of ${\left(\sigma_{Q_{i}Q_{j}}^{}\right)}^{2}$ at the position of *c_T_* = 1640 Ns/m and *k_T_* = 14,100 N/m. The diagram describing the variation of the spectral density function *S_Wc_* corresponding to the vertical displacement *w_c_* at the mid-point of the beam in the frequency range 0–5 rad/s, as shown in Figure 10. The figure shows that the TMD has a great effect on reducing vibrations, removing the ability to resonate in a forced vibration of the structure.

## 5. Conclusions

This paper presents the numerical results of free vibration response problems for beams and space frame systems with TMDs, which combines artificial intelligence models (AI models) to predict the first natural frequency of the structures. In addition, the results of reduction vibration problems with TMDs under a random load described as a stationary process with white noise are also presented. The equations are derived through the finite element method and are verified by comparing them with those from other publications. Based on the computations, the effects of the data set of the TMD on the vibration response of the structures were investigated. The results of this study can be applied in many fields. There is great interest in using AI models to solve mechanical problems, so we decided to use an ANN model to predict natural frequencies because many mechanical problems require natural frequencies as input data in early stages. Applications include the dynamic response problem related to resonance and predicting cracks based on the natural frequencies of structures. Hence, this ANN model can be applied to other mechanical problems. These results are also a good reference for further complicated studies.

## Figures and Tables

**Table 1:** Variation of the first natural frequencies as a function of the *m*_T2_/*m*_0_ ratio.

*m*_T_/*m*_0_ (%)	*c_T_* (Ns/m)	*k_T_* (N/m)	Beam without TMD				Beam with One TMD			
			*f*_1_ (Hz)	*f*_2_ (Hz)	*f*_3_ (Hz)	*f*_4_ (Hz)	*f*_1_ (Hz)	*f*_2_ (Hz)	*f*_3_ (Hz)	*f*_4_ (Hz)
1	100	2000	1.161	5.093	19.933	63.312	0.887	1.178	5.093	19.937
2							0.631	1.171	5.093	19.937
3							0.516	1.169	5.093	19.937
4							0.447	1.169	5.093	19.937
5							0.400	1.169	5.093	19.937
6							0.365	1.169	5.093	19.937
7							0.338	1.168	5.093	19.937
8							0.316	1.168	5.093	19.937
9							0.298	1.168	5.093	19.937
10							0.283	1.168	5.093	19.937

**Table 2:** Variation of the first natural frequencies as a function of *c*_T._

*m*_T_/*m*_0_ (%)	*c_T_* (Ns/m)	*k_T_* (N/m)	Beam without TMD				Beam with One TMD			
			*f*_1_ (Hz)	*f*_2_ (Hz)	*f*_3_ (Hz)	*f*_4_ (Hz)	*f*_1_ (Hz)	*f*_2_ (Hz)	*f*_3_ (Hz)	*f*_4_ (Hz)
10	10	2000	1.161	5.093	19.933	63.312	0.283	1.168	5.093	19.937
	20						0.283	1.168	5.093	19.937
	100						0.283	1.168	5.093	19.937
	150						0.283	1.168	5.093	19.937
	300						0.283	1.168	5.093	19.937
	500						0.283	1.168	5.093	19.937
	700						0.283	1.168	5.093	19.937
	800						0.283	1.168	5.093	19.937
	1000						0.283	1.168	5.093	19.937

**Table 3:** Variation of the first natural frequencies as a function of *k*_T._

*m*_T_/*m*_0_ (%)	*c_T_* (Ns/m)	*k_T_* (N/m)	Beam without TMD				Beam with One TMD			
			*f*_1_ (Hz)	*f*_2_ (Hz)	*f*_3_ (Hz)	*f*_4_ (Hz)	*f*_1_ (Hz)	*f*_2_ (Hz)	*f*_3_ (Hz)	*f*_4_ (Hz)
10	100	100	1.161	5.093	19.933	63.312	0.063	1.161	5.093	19.935
		500					0.142	1.162	5.093	19.936
		1000					0.200	1.164	5.093	19.936
		1500					0.245	1.166	5.093	19.936
		2000					0.283	1.168	5.093	19.937
		3000					0.345	1.172	5.093	19.937
		5000					0.442	1.181	5.093	19.938
		8000					0.552	1.197	5.093	19.940
		10,000					0.611	1.209	5.093	19.941

**Table 4:** Variation of the first natural frequencies as a function of *m*_T2_/*m*_0_ and *m*_T1_/*m*_0_.

*m*_T2_/*m*_0_ (%)	*m*_T1_/*m*_0_ (%)	*c_T_*_1_ (Ns/m)	*k_T_*_1_ (N/m)	Beam without TMD				Beam with Three TMDs			
				*f*_1_ (Hz)	*f*_2_ (Hz)	*f*_3_ (Hz)	*f*_4_ (Hz)	*f*_1_ (Hz)	*f*_2_ (Hz)	*f*_3_ (Hz)	*f*_4_ (Hz)
1	4.0	10^2^	2000	1.161	5.093	19.933	63.312	0.447	0.450	0.888	1.185
1	3.0							0.516	0.519	0.888	1.186
1	2.0							0.631	0.638	0.888	1.187
1	1.0							0.876	0.900	0.901	1.193
2	4.0							0.447	0.450	0.631	1.179
2	3.0							0.512	0.519	0.631	1.179
2	2.0							0.626	0.636	0.637	1.180
2	1.0							0.631	0.888	0.900	1.187
3	3							0.512	0.519	0.520	1.178
3	2							0.516	0.632	0.636	1.179
3	1							0.516	0.888	0.900	1.186
4	3							0.447	0.516	0.519	1.178
4	2							0.447	0.631	0.636	1.179
4	1							0.447	0.888	0.900	1.185
5	2							0.400	0.631	0.636	1.178
5	1							0.400	0.888	0.900	1.185

**Table 5:** Variation of the first natural frequencies as a function of *c*_T1._

*m*_T2_/*m*_0_ (%)	*c_T_*_1_ (Ns/m)	*k_T_*_1_ (N/m)	Beam without TMD				Beam with Three TMDs			
			*f*_1_ (Hz)	*f*_2_ (Hz)	*f*_3_ (Hz)	*f*_4_ (Hz)	*f*_1_ (Hz)	*f*_2_ (Hz)	*f*_3_ (Hz)	*f*_4_ (Hz)
3	10	2000	1.161	5.093	19.933	63.312	0.512	0.519	0.520	1.178
	20						0.512	0.519	0.520	1.178
	100						0.512	0.519	0.520	1.178
	150						0.512	0.519	0.520	1.178
	300						0.512	0.519	0.520	1.178
	500						0.512	0.519	0.520	1.178
	700						0.512	0.519	0.520	1.178
	800						0.512	0.519	0.520	1.178
	1000						0.512	0.519	0.520	1.178

**Table 6:** Variation of the first natural frequencies as a function of *k_T_*_1._

*m*_T2_/*m*_0_ (%)	*c_T_*_1_ (Ns/m)	*k_T_*_1_ (N/m)	Beam without TMD				Beam with Three TMDs			
			*f*_1_ (Hz)	*f*_2_ (Hz)	*f*_3_ (Hz)	*f*_4_ (Hz)	*f*_1_ (Hz)	*f*_2_ (Hz)	*f*_3_ (Hz)	*f*_4_ (Hz)
3	100	100	1.161	5.093	19.933	63.312	0.116	0.117	0.118	1.161
		500					0.259	0.260	0.261	1.164
		1000					0.365	0.367	0.368	1.168
		1500					0.445	0.450	0.451	1.173
		2000					0.512	0.519	0.520	1.178
		3000					0.621	0.636	0.637	1.190
		5000					0.780	0.821	0.822	1.224
		8000					0.924	1.037	1.040	1.306
		10,000					0.976	1.159	1.162	1.382

**Table 7:** The comparison of the first three natural frequencies of the space frame.

Method	$\omega_{i}\;(\mathrm{rad}/s)$		
	$\omega_{1}$	$\omega_{2}$	$\omega_{3}$
SAP-2000	4.437	6.375	6.784
This work	4.426	6.039	6.522

**Table 8:** Variation of the first natural frequencies of the space frame system as a function of *m*_T_/*m*_0_.

*m*_T_/*m*_0_ (%)	*c_T_* (Ns/m)	*k_T_* (N/m)	The Frame System without TMD				The Frame System with One TMD			
			*f*_1_ (Hz)	*f*_2_ (Hz)	*f*_3_ (Hz)	*f*_4_ (Hz)	*f*_1_ (Hz)	*f*_2_ (Hz)	*f*_3_ (Hz)	*f*_4_ (Hz)
1	10^3^	10^4^	0.704	0.961	1.038	1.283	0.475	0.704	0.961	1.038
2							0.336	0.704	0.961	1.038
3							0.274	0.704	0.961	1.038
4							0.237	0.704	0.961	1.038
5							0.212	0.704	0.961	1.038
6							0.194	0.704	0.961	1.038
7							0.179	0.704	0.961	1.038
8							0.168	0.704	0.961	1.038
9							0.158	0.704	0.961	1.038
10							0.150	0.704	0.961	1.038

**Table 9:** Variation of the first natural frequencies of the space frame system as a function of *k*_T_.

*m*_T_/*m*_0_ (%)	*c_T_* (Ns/m)	*k_T_* (N/m)	The Frame System without TMD				The Frame System with One TMD			
			*f*_1_ (Hz)	*f*_2_ (Hz)	*f*_3_ (Hz)	*f*_4_ (Hz)	*f*_1_ (Hz)	*f*_2_ (Hz)	*f*_3_ (Hz)	*f*_4_ (Hz)
10	10^3^	5000	0.704	0.961	1.038	1.283	0.106	0.704	0.961	1.038
		7000					0.126	0.704	0.961	1.038
		8000					0.134	0.704	0.961	1.038
		10,000					0.150	0.704	0.961	1.038
		12,000					0.164	0.704	0.961	1.038
		15,000					0.184	0.704	0.961	1.038
		18,000					0.201	0.704	0.961	1.038
		20,000					0.212	0.704	0.961	1.038
		25,000					0.237	0.704	0.961	1.038

**Table 10:** Variation of the first natural frequencies of the space frame system as a function of *c*_T_.

*m*_T_/*m*_0_ (%)	*c_T_* (Ns/m)	*k_T_* (N/m)	The Frame System without TMD				The Frame System with One TMD			
			*f*_1_ (Hz)	*f*_2_ (Hz)	*f*_1_ (Hz)	*f*_2_ (Hz)	*f*_1_ (Hz)	*f*_2_ (Hz)	*f*_1_ (Hz)	*f*_2_ (Hz)
10	100	10^4^	0.704	0.961	1.038	1.283	0.150	0.704	0.961	1.038
	500						0.150	0.704	0.961	1.038
	800						0.150	0.704	0.961	1.038
	1000						0.150	0.704	0.961	1.038
	1400						0.150	0.704	0.961	1.038
	1600						0.150	0.704	0.961	1.038
	1800						0.150	0.704	0.961	1.038
	2000						0.150	0.704	0.961	1.038
	2500						0.150	0.704	0.961	1.038

**Table 11:** The input and output parameters of this artificial neural network (ANN) model for the beam with three TMDs.

Order	*m_T_*_2_/*m*_0_ (%)	*m_T_*_1_/*m*_0_ (%)	*c_T_* (Ns/m)	*k_T_* (N/m)	Target *f*_1_ (Hz)	Predict *f*_1_ (Hz)
1	1	4.0	100	2000	0.447	**0.447**
2	1	3.0	100	2000	0.516	**0.516**
3	1	2.0	100	2000	0.631	**0.631**
4	1	1.0	100	2000	0.876	**0.876**
5	2	4.0	100	2000	0.447	**0.447**
6	2	3.0	100	2000	0.512	**0.512**
7	2	2.0	100	2000	0.626	**0.626**
8	2	1.0	100	2000	0.631	**0.631**
9	3	3	100	2000	0.512	**0.512**
10	3	2	100	2000	0.516	**0.516**
11	3	1	100	2000	0.516	0.5199
12	4	3	100	2000	0.447	**0.447**
13	4	2	100	2000	0.447	**0.447**
14	4	1	100	2000	0.447	**0.447**
15	5	2	100	2000	0.400	**0.400**
16	5	1	100	2000	0.400	**0.400**
17	3	3	10	2000	0.512	**0.512**
18	3	3	20	2000	0.512	**0.512**
19	3	3	150	2000	0.512	**0.512**
20	3	3	300	2000	0.512	**0.512**
21	3	3	500	2000	0.512	**0.512**
22	3	3	700	2000	0.512	**0.512**
23	3	3	800	2000	0.512	0.5097
24	3	3	1000	2000	0.512	**0.512**
25	3	3	100	100	0.116	**0.116**
26	3	3	100	500	0.259	**0.259**
27	3	3	100	1000	0.365	**0.365**
28	3	3	100	1500	0.445	**0.445**
29	3	3	100	3000	0.621	0.6195
30	3	3	100	5000	0.780	**0.790**
31	3	3	100	8000	0.924	**0.924**
32	3	3	100	10,000	0.976	**0.976**
E_Average_ (%)						**0.0017**
E_max_ (%)						**0.7484**

**Table 12:** The input and output parameters of this ANN model for the space frame system with one TMD.

Order	*m_T_*/*m*_0_ (%)	*c_T_* (Ns/m)	*k_T_* (N/m)	Target *f*_1_ (Hz)	Predict *f*_2_ (Hz)
1	1	1000	10,000	0.475	**0.475**
2	2	1000	10,000	0.336	**0.336**
3	3	1000	10,000	0.274	0.2739
4	4	1000	10,000	0.237	0.2372
5	5	1000	10,000	0.212	0.2121
6	6	1000	10,000	0.194	0.1936
7	7	1000	10,000	0.179	0.1794
8	8	1000	10,000	0.168	0.1678
9	9	1000	10,000	0.158	**0.158**
10	10	1000	10,000	0.150	**0.150**
11	10	1000	5000	0.106	0.1061
12	10	1000	7000	0.126	0.1256
13	10	1000	8000	0.134	0.1343
14	10	1000	12,000	0.164	0.1643
15	10	1000	15,000	0.184	0.1838
16	10	1000	18,000	0.201	0.2013
17	10	1000	20,000	0.212	0.2118
18	10	1000	25,000	0.237	**0.237**
19	10	100	10,000	0.150	**0.150**
20	10	500	10,000	0.150	**0.150**
21	10	800	10,000	0.150	**0.150**
22	10	1400	10,000	0.150	**0.150**
23	10	1600	10,000	0.150	**0.150**
24	10	1800	10,000	0.150	**0.150**
25	10	2000	10,000	0.150	**0.150**
26	10	2500	10,000	0.150	**0.150**
E_Average_ (%)					**0.0073**
E_max_ (%)					**0.3052**

**Figure A1:**
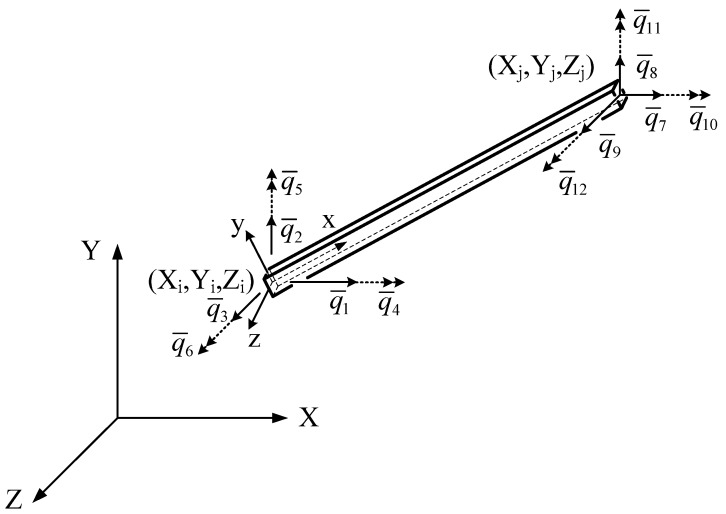
The element nodal displacement components in the global coordinate system.

## Appendix A

### Appendix A.1. The Shape Functions

(A1) $$N_{1u}\left(x\right)=\left(1-\frac{x}{a}\right);\;N_{2u}\left(x\right)=\frac{x}{a}$$

(A2) $$\begin{array}{l} N_{1v}\left(x\right)=\left(1-3\frac{x^{2}}{a^{2}}+2\frac{x^{3}}{a^{3}}\right);\;N_{2v}\left(x\right)=\left(x-2\frac{x^{2}}{a}+\frac{x^{3}}{a}\right);\\ N_{3v}\left(x\right)=\left(3\frac{x^{2}}{a^{2}}-2\frac{x^{3}}{a^{3}}\right);\;N_{4v}\left(x\right)=\left(-\frac{x^{2}}{a^{2}}+\frac{x^{3}}{a^{3}}\right)\\ \frac{dN_{1v}\left(x\right)}{dx}=\left(-6\frac{x}{a^{2}}+6\frac{x^{2}}{a^{3}}\right);\;\frac{dN_{2v}\left(x\right)}{dx}=\left(1-4\frac{x}{a}+3\frac{x^{2}}{a}\right);\\ \frac{dN_{3v}\left(x\right)}{dx}=\left(6\frac{x}{a^{2}}-6\frac{x^{2}}{a^{3}}\right);\;\frac{dN_{4v}\left(x\right)}{dx}=\left(-2\frac{x}{a^{2}}+3\frac{x^{2}}{a^{3}}\right) \end{array}$$

(A3) $$\begin{array}{l} N_{1w}\left(x\right)=\left(1-3\frac{x^{2}}{a^{2}}+2\frac{x^{3}}{a^{3}}\right);N_{2w}\left(x\right)=\left(x-2\frac{x^{2}}{a}+\frac{x^{3}}{a}\right)\\ N_{3w}\left(x\right)=\left(3\frac{x^{2}}{a^{2}}-2\frac{x^{3}}{a^{3}}\right);N_{4w}\left(x\right)=\left(-\frac{x^{2}}{a^{2}}+\frac{x^{3}}{a^{3}}\right)\\ \frac{dN_{1w}\left(x\right)}{dx}=\left(-6\frac{x}{a^{2}}+6\frac{x^{2}}{a^{3}}\right);\frac{dN_{1w}\left(x\right)}{dx}=\left(1-4\frac{x}{a}+3\frac{x^{2}}{a}\right)\\ \frac{dN_{1w}\left(x\right)}{dx}=\left(6\frac{x}{a^{2}}-6\frac{x^{2}}{a^{3}}\right);\frac{dN_{1w}\left(x\right)}{dx}=\left(-2\frac{x}{a^{2}}+3\frac{x^{2}}{a^{3}}\right) \end{array}$$

(A4) $$N_{1\varphi }\left(x\right)=\left(1-\frac{x}{a}\right);N_{2\varphi }\left(x\right)=\frac{x}{a}$$

in which *a* is the length of the frame element.

### Appendix A.2. The Distributed Mass Matrix Components

(A5) $$m_{u}=m_{v}=m_{w}=\left[\begin{array}{cc} m & \\ & m \end{array}\right],m_{\varphi }=\left[\begin{array}{cc} m_{p} & \\ & m_{p} \end{array}\right]$$

### Appendix A.3. The Element Matrices

(A6) $$k=\left[\begin{array}{cccccccccccc} \frac{EF}{a} & 0 & 0 & 0 & 0 & 0 & -\frac{EF}{a} & 0 & 0 & 0 & 0 & 0\\ 0 & \frac{12EI_{z}}{a^{3}} & 0 & 0 & 0 & \frac{6EI_{z}}{a^{2}} & 0 & -\frac{12EI_{z}}{a^{3}} & 0 & 0 & 0 & \frac{6EI_{z}}{a^{2}}\\ 0 & 0 & \frac{12EI_{y}}{a^{3}} & 0 & \frac{6EI_{y}}{a^{2}} & 0 & 0 & 0 & -\frac{12EI_{y}}{a^{3}} & 0 & \frac{6EI_{y}}{a^{2}} & 0\\ 0 & 0 & 0 & \frac{EGJ_{p}}{a} & 0 & 0 & 0 & 0 & 0 & -\frac{EGJ_{p}}{a} & 0 & 0\\ 0 & 0 & -\frac{6EI_{y}}{a^{2}} & 0 & \frac{4EI_{y}}{a} & 0 & 0 & 0 & \frac{6EI_{y}}{a^{2}} & 0 & \frac{2EI_{y}}{a} & 0\\ 0 & \frac{6EI_{z}}{a^{2}} & 0 & 0 & 0 & \frac{4EI_{z}}{a} & 0 & -\frac{6EI_{z}}{a^{2}} & 0 & 0 & 0 & \frac{4EI_{z}}{a}\\ -\frac{EF}{a} & 0 & 0 & 0 & 0 & 0 & \frac{EF}{a} & 0 & 0 & 0 & 0 & 0\\ 0 & -\frac{12EI_{z}}{a^{3}} & 0 & 0 & 0 & -\frac{6EI_{z}}{a^{2}} & 0 & \frac{12EI_{z}}{a^{3}} & 0 & 0 & 0 & -\frac{6EI_{z}}{a^{2}}\\ 0 & 0 & -\frac{12EI_{y}}{a^{3}} & 0 & \frac{6EI_{y}}{a^{2}} & 0 & 0 & 0 & \frac{12EI_{y}}{a^{3}} & 0 & -\frac{6EI_{y}}{a^{2}} & 0\\ 0 & 0 & 0 & -\frac{EGJ_{p}}{a} & 0 & 0 & 0 & 0 & 0 & \frac{EGJ_{p}}{a} & 0 & 0\\ 0 & 0 & \frac{6EI_{y}}{a^{2}} & 0 & \frac{2EI_{y}}{a} & 0 & 0 & 0 & -\frac{6EI_{y}}{a^{2}} & 0 & \frac{4EI_{y}}{a} & 0\\ 0 & \frac{6EI_{z}}{a^{2}} & 0 & 0 & 0 & \frac{4EI_{z}}{a} & 0 & -\frac{6EI_{z}}{a} & 0 & 0 & 0 & \frac{4EI_{z}}{a} \end{array}\right]$$

(A7) $$m=\frac{ma}{420}\left[\begin{array}{cccccccccccc} 140 & 0 & 0 & 0 & 0 & 0 & 70 & 0 & 0 & 0 & 0 & 0\\ 0 & 156 & 0 & 0 & 0 & 22a & 0 & 54 & 0 & 0 & 0 & -13a\\ 0 & 0 & 156 & 0 & 22a & 0 & 0 & 0 & 54 & 0 & -13a & 0\\ 0 & 0 & 0 & \frac{140J_{p}}{F} & 0 & 0 & 0 & 0 & 0 & 70 & 0 & 0\\ 0 & 0 & 22a & 0 & 4a^{2} & 0 & 0 & 0 & 13a & 0 & -3a^{} & 0\\ 0 & 22a & 0 & 0 & 0 & 4a^{2} & 0 & 13a & 0 & 0 & 0 & -3a^{2}\\ 70 & 0 & 0 & 0 & 0 & 0 & 140 & 0 & 0 & 0 & 0 & 0\\ 0 & 54 & 0 & 0 & 0 & 13a & 0 & 156 & 0 & 0 & 0 & -22a\\ 0 & 0 & 54 & 0 & 13a & 0 & 0 & 0 & 156 & 0 & -22a & 0\\ 0 & 0 & 0 & 70 & 0 & 0 & 0 & 0 & 0 & \frac{140J_{p}}{F} & 0 & 0\\ 0 & 0 & -13a & 0 & -3a^{2} & 0 & 0 & 0 & -22a & 0 & 4a^{2} & 0\\ 0 & -13a & 0 & 0 & 0 & -3a^{2} & 0 & -22a & 0 & 0 & 0 & 4a^{2} \end{array}\right]$$

(A8) $$c=\frac{ca}{420}\left[\begin{array}{cccccccccccc} 140 & 0 & 0 & 0 & 0 & 0 & 70 & 0 & 0 & 0 & 0 & 0\\ 0 & 156 & 0 & 0 & 0 & 22a & 0 & 54 & 0 & 0 & 0 & -13a\\ 0 & 0 & 156 & 0 & 22a & 0 & 0 & 0 & 54 & 0 & -13a & 0\\ 0 & 0 & 0 & 140 & 0 & 0 & 0 & 0 & 0 & 70 & 0 & 0\\ 0 & 0 & 22a & 0 & 4a^{2} & 0 & 0 & 0 & 13a & 0 & -3a^{2} & 0\\ 0 & 22a & 0 & 0 & 0 & 4a^{2} & 0 & 13a & 0 & 0 & 0 & -3a^{2}\\ 70 & 0 & 0 & 0 & 0 & 0 & 140 & 0 & 0 & 0 & 0 & 0\\ 0 & 54 & 0 & 0 & 0 & 13a & 0 & 156 & 0 & 0 & 0 & -22a\\ 0 & 0 & 54 & 0 & 13a & 0 & 0 & 0 & 156 & 0 & -22a & 0\\ 0 & 0 & 0 & 70 & 0 & 0 & 0 & 0 & 0 & 140 & 0 & 0\\ 0 & 0 & -13a & 0 & -3a^{2} & 0 & 0 & 0 & -22a & 0 & 4a^{2} & 0\\ 0 & -13a & 0 & 0 & 0 & -3a^{2} & 0 & -22a & 0 & 0 & 0 & 4a^{2} \end{array}\right]$$

### Appendix A.4. The Relationship of the Nodal Displacement Components between the Local Coordinate System and the Global Coordinate System of the Structure

Consider a frame element with two nodes *i* and *j* in any direction in space (Figure A1). Let *xyz* be the local coordinate system of the frame element, and let *XYZ* be the global coordinate system of the structure.

The coordinate components of node *i* and *j* of the frame element in the global coordinate system are *X_i_*, *Y_i_*, and *Z_i_* and *X_j_*, *Y_j_*, and *Z_j_*, respectively. The length of the frame element is then defined as follows:

(A9) $$a=\sqrt{{\left(X_{j}-X_{i}\right)}^{2}+{\left(Y_{j}-Y_{i}\right)}^{2}+{\left(Z_{j}-Z_{i}\right)}^{2}}$$

A matrix indicating the direction of the *xyz* coordinate system versus the *XYZ* coordinate system is of the form

(A10) $$c_{Tr}=\left[\begin{array}{ccc} c_{xX} & c_{xY} & c_{xZ}\\ c_{yX} & c_{yY} & c_{yZ}\\ c_{zX} & c_{zY} & c_{zZ} \end{array}\right]$$

where *c_xX_*, *c_xY_*, and *c_xZ_* are the directional cosines of the *x*-axis relative to the *X-, Y-,* and *Z*-axes, respectively. *c_yX_*, *c_yY_*, and *c_yZ_* are the directional cosines of the *y*-axis relative to the *X-, Y-,* and *Z*-axes, respectively. *c_zX_*, *c_zY_*, and *c_zZ_* are the directional cosines of the *z*-axis relative to the *X-, Y-,* and *Z*-axes, respectively.

For the local coordinate system, the *x*-axis is often seclected as the the main long axis of the frame element, and the *y-* and *z*-axes are the central main axes of the cross section. At that time, the components in Equation (A10) are calculated with the following formula

(A11) $$c_{xX}=\frac{X_{j}-X_{i}}{a},c_{xY}=\frac{Y_{j}-Y_{i}}{a},c_{xZ}=\frac{Z_{j}-Z_{i}}{a}$$

Knowing the location of the central main axes of the *yz*-section, we also know the values of angles β and γ, so we have

(A12) $$c_{zY}=\cos\beta ,c_{yY}=\cos\gamma .$$

Next, four remaining components *c_yX_*, *c_yZ_*, *c_zX_*, and *c_zZ_* of matrix **c** are determined from four familiar expressions in the analytic geometry as follows.

(A13) $$\begin{array}{l} c_{yX}^{2}+c_{yY}^{2}+c_{yZ}^{2}=1,\\ c_{zX}^{2}+c_{zY}^{2}+c_{zZ}^{2}=1,\\ c_{xX}+c_{yX}+c_{xY}+c_{yY}+c_{xZ}+c_{yZ}=0,\\ c_{xX}+c_{zX}+c_{xY}+c_{zY}+c_{xZ}+c_{zZ}=0. \end{array}$$

If we use a numbering principle and a positive-dimensional convention of coordinates in the global coordinate system *XYZ* and in the local coordinate system *xyz* (Figure A1), the matrix **T** transforming from the global coordinate system to the local coordinate system (in the case of the space frame system) has the following form:

(A14) $$T=\left[\begin{array}{cccc} c_{Tr} & 0 & 0 & 0\\ 0 & c_{Tr} & 0 & 0\\ 0 & 0 & c_{Tr} & 0\\ 0 & 0 & 0 & c_{Tr} \end{array}\right]$$

## References

[1] Frahm H. (1909). Device for Damped Vibrations of Bodies. U.S. Patent.

[2] Den Hartog J.P. (1985). Mechanical Vibrations.

[3] Elias S., Matsagar V. (2017). Research developments in vibration control of structures using passive tuned mass dampers. Annu. Rev. Control.

[4] Weisner K.B. (1979). Tuned mass dampers to reduce building wind motion. ASCE Convention and Exposition, Preprint 3510.

[5] Lu Z., Wang D.C., Masri S.F., Lu X.L. (2016). An experimental study of vibration control of wind-excited high-rise buildings using particle tuned mass dampers. Smart Struct. Syst..

[6] Venanzi I. (2015). Robust optimal design of tuned mass dampers for tall buildings with uncertain parameters. Struct. Multidiscipl. Optim..

[7] Kaynia A.M., Veneziano D., John M.B. (1981). Seismic effectiveness of tuned mass dampers. J. Struct. Div..

[8] Tuan A.Y., Shang G.Q. (2014). Vibration control in a 10-storey building using a tuned mass damper. J. Appl. Sci. Eng..

[9] Domizio M.N., Ambrosini D., Curadelli O. (2015). Performance of tuned mass damper against structural collapse due to near fault earthquakes. J. Sound.

[10] Krenk S., Høgsberg J. (2008). Tuned mass absorbers on damped structures under random load. Probabilistic Eng. Mech..

[11] Fujino Y., Abe M. (1993). Design formulas for tuned mass dampers based on A perturbation technique. Earthq. Eng. Struct. Dyn..

[12] Gerges R.R., Vickery B.J. (2005). Optimum design of pendulum-type tuned mass dampers. Struct. Tall Build..

[13] Sun C., Jahangiri V. (2018). Bi-directional vibration control of offshore wind turbines using a 3D pendulum tuned mass damper. Mech. Syst. N.a. Process..

[14] Di Matteo A., Furtmüller T., Adam C., Pirrotta A. (2018). Optimal design of tuned liquid column dampers for seismic response control of base-isolated structures. Acta Mech..

[15] Bigdeli Y., Kim D. (2016). Damping effects of the passive control devices on structural vibration control: TMD, TLC and TLCD for varying total masses. J. Civil Eng..

[16] Lievens K., Lombaert G., Roeck G.D., Van der Broeck P. (2016). Robust design of a TMD for the vibration serviceability of a footbridge. Eng. Struct..

[17] Jiménez-Alonso J.F., Sáez A. (2018). Motion-base ddesign of TMD for vibrating footbridges under uncertainty conditions. Smart Struct. Syst..

[18] Tributsch A., Adam C. (2012). Evaluation and analytical approximation of Tuned Mass Damper performance in an earthquake environment. Smart Struct. Syst..

[19] De Domenico D., Ricciardi G. (2018). Earthquake-resilient design of base isolated buildings with TMD at basement: Application to a case study. Soil Dyn. Earthq. Eng..

[20] De Domenico D., Ricciardi G. (2018). Optimal design and seismic performance of tuned mass damper inerter (TMDI) for structures with nonlinear base isolation systems. Earthq. Eng. Struct. Dyn..

[21] Pietrosanti D., De Angelis M., Basili M. (2017). Optimal design and performance evaluation of systems with Tuned Mass Damper Inerter (TMDI). Earthq. Eng. Struct. Dyn..

[22] De Domenico D., Ricciardi G. (2018). Improving the dynamic performance of base-isolated structures via tuned mass damper and inerter devices: A comparative study. Struct. Control Hlth..

[23] De Domenico D., Ricciardi G. (2018). An enhanced base isolation system equipped with optimal Tuned Mass Damper Inerter (TMDI). Earthq. Eng. Struct. Dyn..

[24] Elias S., Matsagar V., Datta T.K. (2018). Along-wind response control of chimneys with distributed multiple tuned mass dampers. Struct. Heal. Monit..

[25] Hashimoto T., Fujita K., Tsuji M., Takewaki I. (2015). Innovative base-isolated building with large mass-ratio TMD at basement for greater earthquake resilience. Futur. Cities.

[26] What is a Neural Net?. http://www.cormactech.com/neunet/whatis.html.

[27] Issa A. (2007). Computational control of laser systems for micro-machining. Ph.D. Thesis.

[28] Ye J., Yuan X.C., Zhou G. (2003). Genetic algorithm for optimization design of diffractive optical elements in laser beam shaping. Proceed. SPIE.

[29] Xie S.Q. (2005). Optimal process planning for a combined punch-and-laser cutting machine using ant colony optimization. Int. J. Prod..

[30] Shen H., Shi Y.J., Yao Z.Q., Hu J. (2006). Fuzzy logic model for bending angle in laser forming. Mater. Sci. Technol..

[31] De Deus A.M., Mazumder J. (1996). Two-dimensional thermo-mechanical finite element model for laser cladding. J. Laser Appl..

[32] Mohsen O.S., Ali M. (2011). The ANN application in FEM modeling of mechanical properties of Al–Si alloy. Appl. Math. Model..

[33] Esmailzadeh M., Aghaie-Khafri M. (2012). Finite element and artificial neural network analysis of ECAP. Comput. Mater. Sci..

[34] Miguel A., Komal R., Konstantinos D.T., Tiago P.R. (2019). Neural Network-Based Formula for the Buckling Load Prediction of I-Section Cellular Steel Beams. Computers.

[35] Yung H.C., Yen H.H. (2004). Timoshenko beam with tuned mass dampers and its design curves. J. Sound Vib..

